# Removal of Heavy Metal Ions from Wastewaters: An Application of Sodium Trithiocarbonate and Wastewater Toxicity Assessment

**DOI:** 10.3390/ma14030655

**Published:** 2021-01-31

**Authors:** Maciej Thomas, Violetta Kozik, Andrzej Bąk, Krzysztof Barbusiński, Joanna Jazowiecka-Rakus, Josef Jampilek

**Affiliations:** 1 Chemiqua Water & Wastewater Company, Skawińska 25/1, 31-066 Kraków, Poland; 2 Institute of Chemistry, University of Silesia, Szkolna 9, 40-007 Katowice, Poland; andrzej.bak@us.edu.pl; 3 Department of Water and Wastewater Engineering, Silesian University of Technology, Konarskiego 18, 44-100 Gliwice, Poland; krzysztof.barbusinski@polsl.pl; 4 Center for Translational Research and Molecular Biology of Cancer, Maria Skłodowska–Curie National Institute of Oncology–State Research Institute, Wybrzeże AK 15, 44-101 Gliwice, Poland; joanna.jazowiecka@io.gliwice.pl; 5 Department of Analytical Chemistry, Faculty of Natural Sciences, Comenius University, Ilkovicova 6, 84215 Bratislava, Slovakia; josef.jampilek@gmail.com

**Keywords:** galvanic wastewater, heavy metals, sodium trithiocarbonate, *B. plicatilis*, toxicity, central composite design, response surface methodology

## Abstract

The synthesis and application of sodium trithiocarbonate (Na_2_CS_3_) for the treatment of real galvanic wastewater in order to remove heavy metals (Cu, Cd and Zn) was investigated. A Central Composite Design/Response Surface Methodology (CCD/RSM) was employed to optimize the removal of heavy metals from industrial wastewater. Adequacy of approximated data was verified using Analysis of Variance (ANOVA). The calculated coefficients of determination (*R*^2^ and *R*^2^_adj_) were 0.9119 and 0.8532, respectively. Application of Na_2_CS_3_ conjugated with CCD/RSM allowed Cu, Cd and Zn levels to be decreased and, as a consequence, ∑_Cu,Cd,Zn_ decreased by 99.80%, 97.78%, 99.78%, and 99.69%, respectively, by using Na_2_CS_3_ at 533 mg/L and pH 9.7, within 23 min. Implementation of conventional metal precipitation reagents (NaOH, Ca(OH)_2_ and CaO) at pH 11 within 23 min only decreased ∑_Cu,Cd,Zn_ by 90.84%, 93.97% and 93.71%, respectively. Rotifer *Brachionus plicatilis* was used to conduct the assessment of wastewater toxicity. Following the application of Na_2_CS_3_, after 60 min the mortality of *B. plicatilis* was reduced from 90% to 25%. Engagement of Na_2_CS_3_ under optimal conditions caused the precipitation of heavy metals from the polluted wastewater and significantly decreased wastewater toxicity. In summary, Na_2_CS_3_ can be used as an effective heavy metal precipitating agent, especially for Cu, Cd and Zn.

## 1. Introduction

Heavy metal salts are widely used throughout industry. Rapid transformation of traditional societies into industrial ones, the introduction of mechanization-based production, effective planning and management methods have resulted not only in improved effectiveness and quality of production processes, but have also exerted a negative impact on the natural environment. Due to such intensified industrialization trends, heavy metals from various branches of industry are released into ecosystems at a constantly increasing rate. As a consequence, metals such as Cd, Cr, Cu, Hg, Ni, Pb and Zn are detected in industrial wastewater from electroplating facilities, mining and metallurgy plants, tanning and petroleum industries, paint and pigment production sites, etc. [1,2].

Heavy metals are defined as metals characterized by a specific density of >5 g/cm^3^ and, in addition, which have an adverse impact on the natural environment including living organisms [3]. On the one hand, some metal cations at low physiological concentrations play important functions contributing to proper functioning of plants, animals and humans. On the other hand, at high concentrations that exceed some predefined threshold values, they are toxic and disrupt normal physiological processes. Due to their toxicity, persistence, as well as environmental mobility, heavy metals are one of the most problematic air, water and soil pollutants in the context of food production and ecological issues as well as evolutionary aspects [4,5].

The problems concerning toxicity of heavy metals should be considered individually for each metal, due to their unique physicochemical properties, which imply different mechanisms of interaction with cells and tissues of living organisms. Unfortunately, these mechanisms are not yet fully elucidated. It has been clearly demonstrated that heavy metal cations can variously affect cell organelles including cell membrane, lysosomes, endoplasmic reticulum as well as the cell nucleus. They can also modify the activity of certain enzymes that play important roles in metabolism, detoxification or damage repair due to toxic substances [6]. Moreover, heavy metal cations can interact with DNA and nuclear proteins and thus contribute to the formation of significant conformational changes, DNA damage and, as a result, to the processes of carcinogenesis and apoptosis [7,8,9]. Formation of Reactive Oxygen Species (ROS) in the cells of living organisms and the phenomenon of oxidative stress have a significant impact on the degree of toxicity and carcinogenicity of heavy metal cations, as was shown for As [10], Cd [11], Cr [12], Pb [13] and Hg [14], among others.

Chemical and electrochemical metal deposition processes are engaged in many industrial plants for technical and decorative purposes and are preceded by several preliminary operations such as cleaning, degreasing, etching and activation, respectively. The purpose of these preliminary processes is to properly prepare the workpiece surface *prior to* covering with a metal layer. These operations and processes generate wastewater that contains heavy metal cations. Such wastewater must be treated when it is reused in production processes or before discharging into sewage systems or water courses.

Untreated galvanic wastewater is very toxic due to the presence of cyanides (CN^−^), hexavalent chromium (Cr^6+^) and heavy metals such as Cu, Zn, As, Be, Cd, Pb, Ni, etc. [15,16,17]. Additionally, galvanic wastewater contains various concentrations of metals, which depends on the type of workpiece subjected to treatment (e.g., made of steel, brass, etc.), the type of process they come from (e.g., electrochemical copper plating, final surface finishing operations, etc.), type of metallization technology used (chemical, electrochemical processes, etc.), the type of rinsing methods implemented (cascade rinsing, intermittent scrubbers, etc.) and the employment of certain recovery and reuse methods in the rinsing water (ion exchange methods, membrane methods, etc.), among others.

Example data show that wastewater from the chrome plating process (pH = 4) contained 0.105 mg/L Cu, 24.53 mg/L Cr, 3.380 mg/L Ni, 7.528 mg/L Zn and 1.188 mg/L Pb. Galvanic wastewater (pH = 4) from electrochemical processes using cyanide baths contained (apart from CN^−^ ions) 5.194 mg/L Cu, 2.113 mg/L Cr, 35.56 mg/L Ni, 75.86 mg/L Zn and 0.013 mg/L Pb, while the acid–alkaline wastewater from washing processes contained small amounts of heavy metals—i.e., 0.621 mg/L Cu, 0.240 mg/L Cr, 2.970 mg/L Ni, 4.810 mg/L Zn and 0.025 mg/L Pb [18]. On the other hand, the wastewater from chemical and electrochemical processing of printed circuit boards (PCBs) revealed different concentrations of copper, depending on the type of the derived process—i.e., 3–20 mg/L (after alkaline and acid etching processes), 0.1–0.5 mg/L (after chemical copper plating), 0.5–3.0 mg/L (after electrolytic copper plating) and 10–60 mg/L (after brushing) [19]. Wastewater from the manufacturing of PCBs was characterized by pH and COD (Chemical Oxygen Demand) in the range of 2.4–9.6 and 12–280 mg O_2_/L (after chemical and electrochemical copper plating), 1.8–2.5 and 74–577 mg O_2_/L (after acid etching), 10.2–13.0 and 10.860–25.680 mg O_2_/L (after photopolymer development and stripping) and 7.6–7.8 and 113–364 mg O_2_/L (after brushing). For the above processes, copper concentrations in the wastewater ranged from 9–91 mg/L, 405–919 mg/L, 11–147 mg/L and 72–230 mg/L, respectively [20]. Additionally, the analysis of the influence of wastewater containing copper compounds (1200 mg/L) from the alkaline etching processes on the respiratory properties of the activated sludge (the so-called respirometric analysis) showed 100% inhibition of the respiratory properties of the activated sludge [21]. On the other hand, the wastewater from the continuous electrochemical tinning of copper wires was strongly acidic (pH < 2) and contained: 3100 mg/L Sn, 27.6 mg/L Fe, 2.41 mg/L Ni and 1.46 mg/L Pb [22] among others.

Wastewater from electroplating processes requires the use of effective treatment methods in order to precipitate heavy metals (and other pollutants) and, consequently, minimize their potential negative impact on the natural environment. For the treatment of galvanic wastewater, a number of methods of varying effectiveness are employed, such as conventional methods of chemical precipitation of metals (including coagulation, sedimentation and flocculation of the formed sediments), in the form of hydroxides (using NaOH, Ca(OH)_2_ etc.) [23], sulfides (using Na_2_S, NaHS, FeS to generate H_2_S at pH < 3, etc.) [24], as well as combined methods involving the use of chemical methods in the first stage, followed by nanofiltration or ion exchange [25,26]. There are also processes that use the phenomenon of adsorption on activated carbon [27], low-cost adsorbents, e.g., lignin [28], diatomite [29], zeolites [30] and biosorbents, obtained from nonliving biomass (bark, lignin, shrimp, krill etc.), algal biomass and microbial biomass (e.g., bacteria, fungi and yeast) [31]. Membrane methods are also applied, including micellar enhanced ultrafiltration (MEUF), polymer enhanced ultrafiltration (PEUF) [32,33], reverse osmosis (RO) [34] and nanofiltration (NF) [35]. Electrochemical methods such as electrocoagulation (EC) are also used to remove heavy metals from wastewater [36]. 

Chemical precipitation methods are usually effective and are widely used due to the possibility of applying simple technological systems and low costs of wastewater treatment. However, the effectiveness of chemical methods decreases in the case of wastewater containing complex compounds of heavy metals. In these cases, highly effective organic or inorganic precipitants, such as sodium diethyl- and dimethylodithiocarbamate (NaS_2_CN(C_2_H_5_)_2_, NaS_2_CN(CH_3_)_2_, respectively), trimercapto-s-triazine, trisodium salt (C_3_N_3_S_3_Na_3_) and sodium trithiocarbonate (Na_2_CS_3_) are applied [37,38]. Sodium diethyl- and dimethylodithiocarbamate and trimercapto-s-triazine trisodium salts are widely used at an industrial scale, while Na_2_CS_3_ is less frequently employed, despite the fact that it is equally effective, and the produced metal trithiocarbonate deposits are characterized by very good sedimentation and filtration properties [39]. So far, its use (also at an industrial scale) for the treatment of industrial wastewater from the production of PCBs containing Cu, Ni and Sn, in the presence of complexing compounds such as Na_2_EDTA (ethylenediaminetetraacetic acid, disodium salt), NH_3(aq)_, NH_2_-CS-NH_2_ (thiourea), Na_3_MGDA (methylglycinediacetic acid, trisodium salt), Na_4_GLDA (N,N-dicarboxymethyl glutamic acid, tetrasodium salt) [37,38,39] and for the precipitation of Rare Earth Elements (REEs) from Acid Mine Drainage (AMD) [40]. The wide application possibilities of Na_2_CS_3_ result from patents [41]; however, there are no scientific investigations presenting the engagement of Na_2_CS_3_ for the treatment of real galvanic wastewater, containing metals other than copper, nickel and tin, in various concentrations and the assessment of the toxicity of treated wastewater after the usage of the precipitant. This is necessary to investigate the potential and effectiveness of this precipitating agent when used to remove metals from a particularly complex matrix such as galvanic wastewater.

As a matter of fact, the paper presents a method for the synthesis of Na_2_CS_3_ from Na_2_S and CS_2_ and the optimization of its use for the removal of a mixture of Cu, Cd and Zn cations from real galvanic wastewater originating from a manufacturing plant where copper, cadmium and zinc plating are used. Additionally, Central Composite Design/Response Surface Methodology (CCD/RSM) was implemented to optimize the metal removal process and the toxicity of untreated and treated wastewaters was assessed using the rotifer *B. plicatilis*.

## 2. Materials and Methods

### 2.1. Chemicals and Synthesis of Sodium Trithiocarbonate (Na_2_CS_3_)

All reagents, unless otherwise stated, were of analytical grade (Avantor^TM^), Gliwice, Poland). For pH adjustment, 10% and 20% NaOH and H_2_SO_4_ solutions (Avantor^TM^) and 15% suspension of CaO (CaO + MgO ≥ 91%, highly reactive burned lime, technical grade, and Ca(OH)_2_ ≥ 93%, technical grade (Trzuskawica, Nowiny, Poland) were used. A 0.10% solution of Praestol 2640 anionic flocculant in distilled water (Solenis^TM^, Capelle aan den IJssel, Holland) was used for flocculation of the precipitates. The artificial sea water contained 58.490% NaCl, 26.460% MgCl_2_·6H_2_O, 9.750% Na_2_SO_4_, 2.765% CaCl_2_, 1.645% KCl, 0.477% NaHCO_3_, 0.238% KBr, 0.071% H_3_BO_3_, 0.095% SrCl_2_·6H_2_O, 0.007% NaF and distilled water, prepared as described previously [42]. Additionally, deionized water (<2 µS/cm) was used to prepare and dilute the solutions. The synthesis of the Na_2_CS_3_ solution was carried out by a modified method, based on the information on the synthesis of K_2_CS_3_ [43] and Na_2_CS_3_ [43,44,45,46,47]. Na_2_S was used for synthesis (approx. 60% Na_2_S, ≤2% Na_2_SO_3_, ≤0.004% of substances insoluble in water, ≤2% Na_2_S_2_O_3_, ≤2% Na_2_CO_3_, ≤0.001% Fe, technical grade, Brenntag, Kędzierzyn-Koźle, Poland), CS_2_ (99.9% CS_2_, technical grade, Siarkopol, Grzybów, Poland) and saturated NaOH solution (approx. 50%) (Avantor^TM^). The synthesis of Na_2_CS_3_ was carried out in such a way that 50 mL of water (2.77 mol) and 37 g of CS_2_ (0.49 mol) were introduced into a three-necked flask (500 mL) equipped with a reflux condenser, stirrer, dropping funnels and nitrogen inlet (technical grade, Air Liquide, Kraków, Poland) and was vigorously stirred, keeping the temperature of the mixture at a maximum of 30–35 °C. Then, 5.7 g of Na_2_S (0.07 mol) was added every hour—a total of 45.6 g (0.58 mol). Then, 1.62 g (0.04 mol) of NaOH as an approx. 50% saturated solution was carefully introduced. The prepared reaction mixture was stirred for eight hours and then left to separate the phases. The lower layer constituting the Na_2_CS_3_ solution was separated and filtered through a fritted funnel to remove solid impurities, and selected physicochemical parameters of the product obtained were determined using the methods described in Section 2.4.

### 2.2. Origin and Physicochemical Parameters of Galvanic Wastewater

In this study, galvanic wastewater originated from an industrial plant located in eastern Poland was used. In the electroplating plant, chemical and electrochemical copper plating, cadmium plating and zinc plating processes are applied. Unit samples were taken from the storage tank collecting all raw industrial wastewater generated in the plant, and averaged by mixing *prior to* pumping into reactors where treatment processes are carried out. One-liter unit samples were taken manually, every hour during a 24-h period. The average sample used in the tests was obtained by mixing unit samples. The average daily sample was not fixed and until the tests were performed, it was stored at 4 °C. Basic physicochemical parameters such as pH, specific conductivity, salinity, content of complex compounds (expressed as EDTA) and heavy metal concentrations (Cu, Cd, Zn) were determined in the wastewater sample using the methods described in Section 2.4.

### 2.3. Apparatus and Experiment Conditions

All experiments were conducted at a constant temperature (19 ± 1 °C) in beakers containing 1000 ± 5 mL of wastewater, which during the research was mixed with a magnetic stirrer at a constant speed of 250 rpm (at the metal precipitation stage) and 50 rpm for 1 min at the stage of flocculation of precipitated sediments. The research was carried out in such a way that 10% or 20% NaOH solutions were added to 1000 ± 5 mL of wastewater in order to adjust the pH to the value assumed in the experimental plan; then, the assumed volume of 40.8% Na_2_CS_3_ solution (corresponding to the amount of pure Na_2_CS_3_ presented in the experimental plan) was added using a micropipette and the pH was corrected again to the assumed value with 10% or 20% H_2_SO_4_ solution and precipitation was carried out for the assumed time. Next, the precipitates were flocculated with 1.0 mL of 0.1% Praestol 2640 (anionic flocculant) solution and, after mixing (1 min/50 rpm), the precipitates were sedimented for 30 min. After this time, samples of treated wastewater were collected from above the sludge, filtered through a 0.45 µm membrane filter and subjected to the tests described in Section 2.4. For the most favorable metal removal conditions determined, a verification experiment was performed and the wastewater was subjected to the physicochemical and toxicological tests described in Section 2.4. Tests using conventional precipitants (20% NaOH, 15% Ca(OH)_2_ suspension or CaO) were conducted as above except that 1000 ± 5 mL of wastewater was alkalized to pH = 11 with the selected reagent.

### 2.4. Analytical Procedures

The Na_2_CS_3_ content (%) in the synthesized solution was determined by the modified method presented previously [48], which consisted of calculating the percentage of Na_2_CS_3_ content as the difference between the total content of reducing substances and the content of reducing impurities (SO_3_^2−^ and S_2_O_3_^2−^), The total content of reducing substances was determined by introducing the test sample into the standard I_2_ solution and back-titrating the excess I_2_ with the standard Na_2_S_2_O_3_ solution against starch as an indicator. The content of reducing admixtures was determined by direct titration of the sample with standard I_2_ solution after prior separation of S^2−^ and CS_3_^2−^ in the form of ZnS and ZnCS_3_. Density of Na_2_CS_3_ at 19 °C was determined using the standard pycnometric method. The pH values, salinity and temperature were measured using an Inolab^®^ pH/Ion/Cond/Temp 750 m and SenTix^®^ 81 electrodes (WTW, Weilheim in Oberbayern, Germany) [49]. The content of complex compounds expressed as EDTA (mg/L) was determined with the Nanocolor^®^ Organic Complexing Agents test kit (Macherey Nagel, Düren, Germany) by using photometric determination through decoloration of the bismuth xylenol orange complex [50]. The content of Cu, Cd and Zn was determined using ICP-OES in accordance with ISO 11885: 2007 [51].

To determine the toxicity of untreated and treated wastewaters (after neutralization of samples to pH 7–7.5) using Na_2_CS_3_ under the most favorable conditions and NaOH, 15% suspension of Ca(OH)_2_ and CaO, a test was performed using a method described previously [52,53,54] with modifications, using neonates of rotifer *B. plicatilis*. The organisms were incubated at 25 °C for 15, 30, 45 and 60 min in the dark. Synthetic sea water (9.0 mL), untreated or treated wastewater tested (1.0 mL) and 10 neonates of rotifer *B. plicatilis* were introduced into sterile glass vials. The control sample contained 10.0 mL of synthetic seawater. After an incubation period of 15, 30, 45 and 60 min, the number of living and dead organisms in each culture was calculated using the Eclipse E200–LED trinocular microscope (Nikon Instruments Europe B.V., Amsterdam, The Netherlands) and reported as the rotifer mortality percent.

### 2.5. Optimization of the Experiments

Central Composite Design (CCD) and Response Surface Methodology (RSM) were used to optimize the process of Cu, Cd and Zn precipitation from the tested wastewater. Literature data show that the lowest concentration of metal ions in the treated wastewater following precipitation of Cu(OH)_2_, Cd(OH)_2_ and Zn(OH)_2_ was obtained at pH = 9.1, 11.2 and 9.2, respectively. In the case of sulfide precipitation, the most favorable pH values for the precipitation of CuS, CdS and ZnS are approx. 6.5, 11 and 11, respectively [55]. Therefore, it was assumed that some metal ions would precipitate as metal hydroxides as a result of wastewater alkalization, which would probably be beneficial regarding the reduction in the stoichiometric amount of Na_2_CS_3_ (497 mg/L of wastewater, corresponding to 0.376 mL of 40.8% Na_2_CS_3_) required for complete precipitation of metals. At the same time, Polish requirements for the pH of wastewater discharged into sewage disposal systems are set at 6.5–9.5 [56]. The adopted precipitation pH value should therefore take into account these requirements and ensure that, as far as possible, there is no need to readjust the pH of the wastewater.

Based on the presented data and several preliminary experiments, it was assumed that three independent parameters would be optimized—i.e., pH, Na_2_CS_3_ dose (mg) and reaction time (min). The sum of metal concentrations, i.e., ∑_Cu,Cd,Zn_ (mg/L), after the wastewater treatment process with Na_2_CS_3_ was adopted as a dependent parameter. The following ranges of independent parameters were finally adopted: pH 7–9, Na_2_CS_3_ dose 300–500 mg and reaction time 15–30 min. CCD was used to plan the experiments and the corresponding input parameters were obtained for 16 experiments, as shown in Table 1. Sixteen experiments were performed (in triplicate). The wastewater tests were carried out as described in Section 2.3 and the values of the ∑_Cu,Cd,Zn_ (mg/L) parameter were determined for each of the experiments. The obtained experimental results were statistically analyzed using the Statistica 13 package (StatSoft, Kraków, Poland) in order to determine the influence of the independent parameters (pH, Na_2_CS_3_ dose (mg) and the reaction time (min)) on the value of the dependent parameter—i.e., ∑_Cu,Cd,Zn_ (mg/L). The relationships are presented using the response surface plots. For the most favorable conditions, the model was verified experimentally and toxicity tests were also performed.

## 3. Results and Discussion

### 3.1. Physicochemical Parameters of Synthesized Na_2_CS_3_ Solution

Table 2 shows the selected physicochemical properties of sodium trithiocarbonate solution obtained by direct synthesis from Na_2_S and CS_2_.

We used the direct reaction of Na_2_S with CS_2_ under synthesis conditions, with the formation of Na_2_CS_3_ in accordance with Equation (1):

(1) $${\mathrm{Na}}_{2}S\;+\;{\mathrm{CS}}_{2}\;\to \;{\mathrm{Na}}_{2}{\mathrm{CS}}_{3}$$

Due to the fact that technical grade Na_2_S containing NaHS was used for the synthesis, in the second stage approximately 50% saturated NaOH solution was added in order to convert the NaHS present in the reaction medium into an additional amount of Na_2_S according to Equation (2):

(2) $$\mathrm{NaHS}\;+\;\mathrm{NaOH}\;\to \;{\mathrm{Na}}_{2}S\;+\;H_{2}O$$

Since the density of the resulting Na_2_CS_3_ solution is greater than that of CS_2_, accumulation of excess CS_2_ on the surface of the Na_2_CS_3_ solution was observed. The obtained Na_2_CS_3_ solution was characterized by an intense, dark red color and a strongly alkaline reaction (pH > 13). Previously conducted studies confirmed that trithiocarbonates have the form of yellow or red-brown crystals, that are well soluble in water, and their solutions are intensely red [43,45,47].

Various methods of determining trithiocarbonates are known from the literature, e.g., with application one-step titration wit potassium ferricyanide, using Fe(II)–dimethylglyoxyme or sodium nitroprusside as indicator [57], direct titrimetric in presence of sulfite, thiosulfate and thiocyanate by titration with o-hydroxymercuribenzoate with sodium nitroprusside as indicator [58] (in both methods, sulfides are determined together with trithiocarbonates) or a method with the separation of sulfur compounds by means of a hexane solution of tributhyl tin (TBT) followed with o-hydroxymercuribenzoic acid titration in the presence of dithizone as indicator [59]; however, this is a multistage, time-consuming method and requires application of KCN in addition to TBT. Due to the fact that the reaction efficiency is practically 100% with respect to the sum of sulfides (Na_2_S and NaHS) [44] and the use of an excess of CS_2_, a simplified method was employed to determine the concentration of Na_2_CS_3_ that is based on determining the concentration of Na_2_CS_3_ as the total content of reducing substances (Na_2_CS_3_ + SO_3_^2−^ + S_2_O_3_^2−^) decreased by the concentration of reducing admixtures (SO_3_^2−^ and S_2_O_3_^2−^) [48]; the method is quite sufficient to determine the concentration of Na_2_CS_3_ in solution in the context of its further use in wastewater treatment. According to this method, a 40.8% Na_2_CS_3_ solution was obtained, which was utilized in the presented research.

### 3.2. Physicochemical Parameters of Galvanic Wastewater

Table 3 presents selected physicochemical parameters of the actual galvanic wastewater used in the investigation.

The tested wastewater was acidic (pH = 3.4) and was characterized by a high electrical conductivity (EC) and salinity value (25,800 µS/cm and 12,850 mg NaCl/L, respectively). Additionally, it contained complexing compounds, determined as EDTA (180 mg/L) and heavy metal ions such as Cu^2+^, Cd^2+^ and Zn^2+^, with the highest concentration of copper (59 mg/L). Research reports indicate that the values of pollution parameters for galvanic wastewater vary in a wide range [18,19,20,22], both in terms of qualitative and quantitative compositions, which depend on technical and technological factors. The reported studies indicate that the concentration of copper in galvanic wastewater can be as high as 30,000 mg/L [60], while that of zinc reaching 1.392 mg/L at low concentrations of copper (0.74 mg/L), nickel (1.37 mg/L), chromium (0.28 mg/L) and iron (1.91 mg/L) [61]. In addition, the study of wastewater from PCB production showed that it contained 20.9 and 128.5 mg/L chelating agents determined as Na_2_EDTA [37,38]. In the case of the tested wastewater (180 mg/L as EDTA), the type of complexing compound was not known, but considering the chelating compounds most commonly used in electroplating, it can be assumed that EDTA, NTA and some organic acids were present—e.g., succinic acid [62].

### 3.3. CCD/RSM Results

Sodium trithiocarbonate (Na_2_CS_3_) is a compound that is highly soluble in water and therefore undergoes electrolytic dissociation, and as a salt of a strong base and weak trithiocarbonic acid (H_2_CS_3_). In addition, it also undergoes hydrolysis and, additionally, in the presence of oxygen from the air, it decomposes very slowly, according to Equations (3)–(5):

Na_2_CS_3(aq)_ → 2Na ^+^ + CS_3_^2−^ (3)

Na_2_CS_3_ + 3H_2_O → Na_2_CO_3_ + 3H_2_S↑ (4)

2Na_2_CS_3_ + 2H_2_O + 2O_2_ → Na_2_CO_3_ + Na_2_S_2_O_3_ + CS_2_↑ + 2H_2_S↑ (5)

Moreover, it should be noted that as a result of wastewater alkalization (by adding NaOH and Na_2_CS_3_), precipitation of sparingly soluble metal hydroxides, i.e., Cu(OH)_2_, Cd(OH)_2_ and Zn(OH)_2_ will occur. Therefore, taking into account the specific properties of Na_2_CS_3_ (Equations (3), (4), and, under certain conditions, Equation (5)), it should be concluded that the precipitated sludge will be a mixture of mainly sparingly soluble metal hydroxides, trithiocarbonates and sulfides, in accordance with Equations (6)–(8):

Me^2+^ + 2OH^−^ → Me(OH)_2_↓ (6)

Me^2+^ + S^2−^ → MeS↓ (7)

Me^2+^ + CS_3_^2−^ → MeCS_3_↓ (8)

Table 1 shows the results of 16 experiments performed for various pH values, Na_2_CS_3_ doses and reaction times. The analysis of the presented experimental data shows that the lowest values of ∑_Cu,Cd,Zn_ were obtained for experiments with a more alkaline reaction environment and a higher dose of Na_2_CS_3_ than in the remaining experiments (Equations (7), (8) and (12)). For Equations (7) and (8) (pH 10, Na_2_CS_3_ 500 mg/L), which differed only in the reaction time (10 and 30 min, respectively), a slightly lower value of ∑_Cu,Cd,Zn_ was obtained with extended reaction times (0.68 and 0.57 mg/L, respectively). This may suggest that for the effective precipitation of metals it is necessary to carry out the precipitation reaction in a certain sufficiently long time. Table 4 presents statistical evaluation of the independent parameters (pH, Na_2_CS_3_, time) and their influence on the value of the dependent parameter (∑_Cu,Cd,Zn_).

Moreover, a high value of the corrected coefficient of determination (85.32%) indicates a high degree of model fit to another sample of data from the same population. The obtained values (*R*^2^) are comparable to the other available data regarding heavy metal removal from real power plant wastewater using electrocoagulation (*R*^2^ = 99.5% and *R*^2^ = 99.6% for iron and nickel, respectively) [63], biosorption of Hg (II) ions from aqueous media by *Polyporus squamosus* (*R*^2^ = 92.4%) [64] and optimization of coagulation–flocculation process for wastewater originating from automotive industry and containing Fe, Cr, Cu ions (*R*^2^ = 92.08%, *R*^2^ = 93.62%, *R*^2^ = 71.87% for Fe, Cr, Cu removal, respectively) [65] and also for organic substances (*R*^2^ = 0.8477, *R*^2^_adj_ = 0.7462) [66,67]. Table 5 presents the results of the model adequacy verification using an analysis of variance.

**Figure 1:**
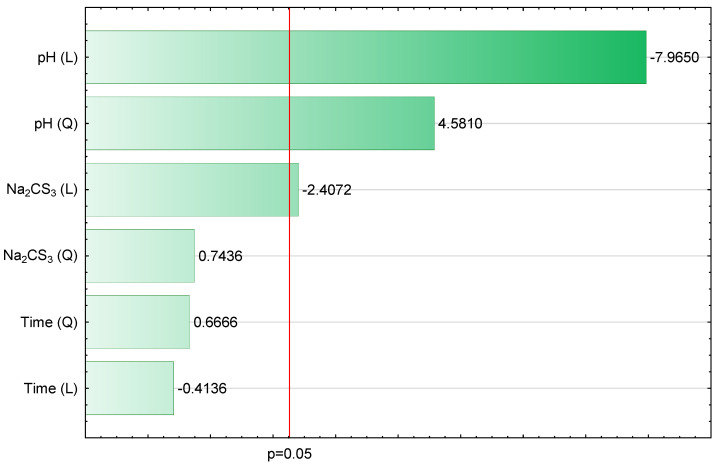
Bar chart of standardized effects (∑_Cu,Cd,Zn_, mg/L, *R*^2^ = 0.9119, *R*^2^*_adj_* = 0.8532, 3 Parameters, 1 Block, 16 Experiments, MS = 0.1219, L—linear effect, Q—quadratic effect and *p*—the absolute value of the standardized effect evaluation).

The obtained results indicate that three out of the six analyzed independent parameters are statistically significant, which means that they have a significant impact on the value of the investigated dependent parameter (∑_Cu,Cd,Zn_). These data correspond with the bar chart of standardized effects presented in Figure 1.

**Figure 2:**
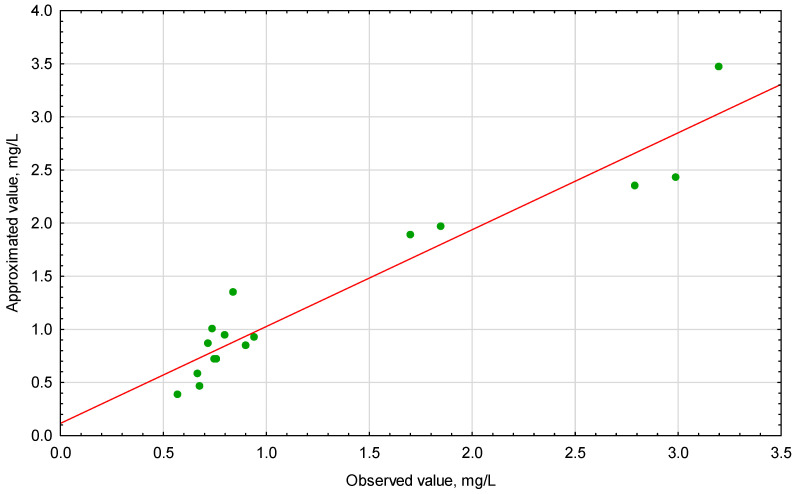
Approximated versus observed values plots for ∑_Cu,Cd,Zn_, mg/L.

Figure 1 is a visual representation of the model validation and shows estimates of standardized effects grouped by their absolute values. The lengths of the horizontal bars are proportional to the absolute value of the standardized effects, while the vertical line indicates the absolute value (*p* = 0.05) of the standardized effect evaluation. The statistically significant parameters show absolute values above the adopted significance level. The positive and negative signs describe the cases where the value of ∑ _Cu,Cd,Zn_ is enhanced or weakened, respectively, when going from the lowest to the highest level set for a specific variable. Therefore, it should be assumed that the values of ∑_Cu,Cd,Zn_ are most influenced (in descending order) by pH (L), pH (Q) and Na_2_CS_3_ (L). Accordingly, Na_2_CS_3_ (Q), Time (Q) and Time (L) have an inconsiderable impact on the ∑_Cu,Cd,Zn_ value. Figure 2 shows the relationship between the observed values and those approximated from the model.

**Figure 3:**
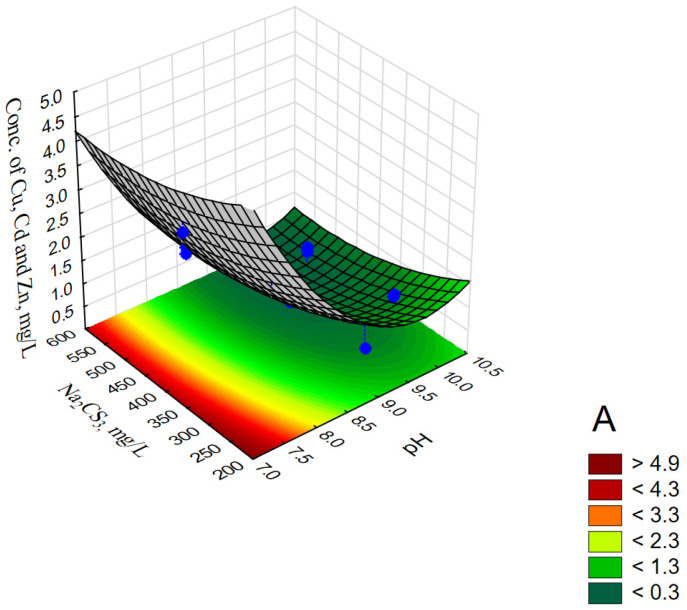

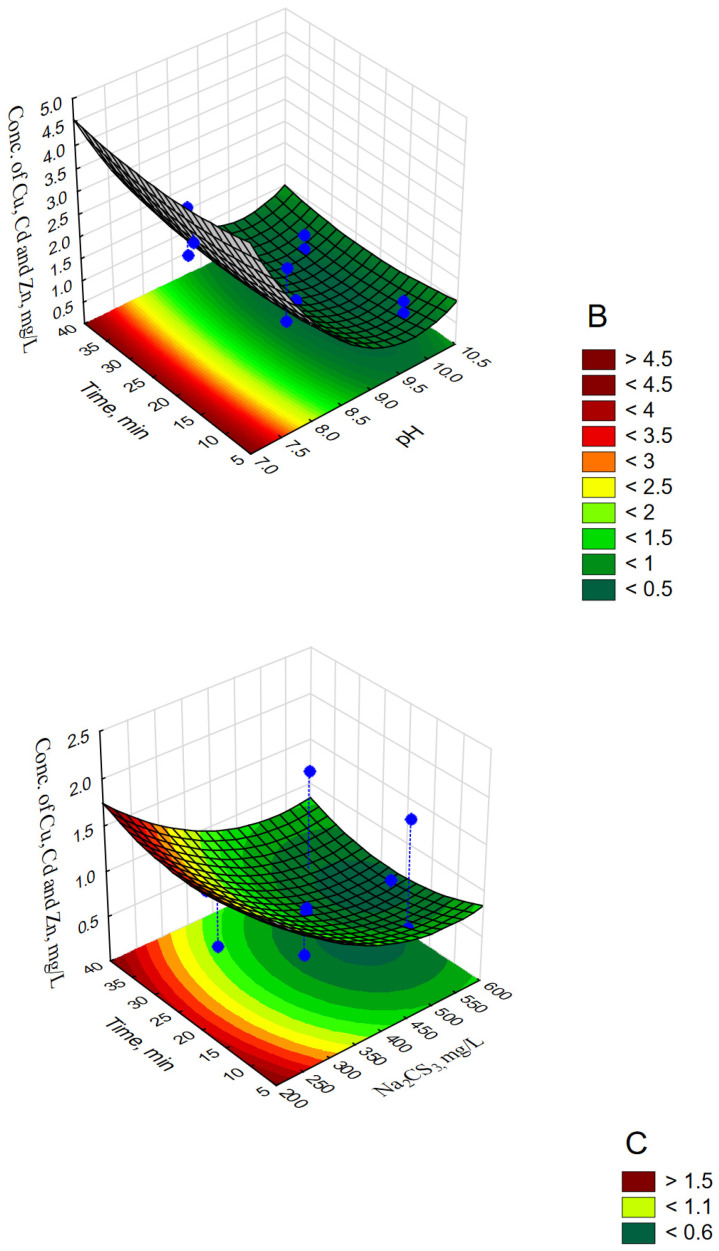
Response surface plots for ∑_Cu,Cd,Zn_, mg/L with respect to pH and Na_2_CS_3_, mg/L (**A**); pH and Time, min (**B**); Na_2_CS_3_, mg/L and time, min (**C**).

The analyzed data showed a linear relationship between the observed values and the values approximated from the model. Moreover, the points reflecting the experimental data are generally randomly distributed around the estimated relationship, which proves the adequacy of the model. Figure 3A–C illustrates the response surface plots for ∑_Cu,Cd,Zn_ with respect to pH, Na_2_CS_3_ concentration and Time.

The performed model tests (CCD/RSM) showed that at a constant reaction time (Time 20 min), the smallest values (<0.3 mg/L) of ∑_Cu,Cd,Zn_ were obtained at pH 9.3–10.3 and Na_2_CS_3_ concentration > 400 mg/L (see Figure 3A). For a constant precipitant concentration (Na_2_CS_3_ concentration 400 mg/L), the lowest values (<0.5 mg/L) of ∑_Cu,Cd,Zn_ were obtained at pH 9.25–10.1 during 17–33 min (see Figure 3B). Similar values for the reaction time at constant pH 9 and Na_2_CS_3_ concentration in the range of 490–600 mg/L (see Figure 3C) made it possible to obtain values of ∑_Cu,Cd,Zn_ < 0.6 mg/L.

The analysis of the effect of wastewater pH on the value of ∑_Cu,Cd,Zn_ and, therefore, on the concentration of individual metals, suggests that excessive increase in the pH value in the case of the tested wastewater may be caused by an increase in the concentration of metals. This phenomenon may result both from the presence of amphoteric hydroxides (Zn(OH)_2_, Cu(OH)_2_) in the tested wastewater [55,66], as well as from the increased solubility of complexes (or salts formed in the presence of other substances in the wastewater, e.g., NH_4_^+^) of the CS_3_^2−^ ion with metals at pH > 10—e.g., [Cu(CS_3_)_n_]^n−^, [Zn(CS_3_)_n_]^n−^, [Cd(CS_3_)_n_]^n−^ KCuCS_3_, NH_4_CuCS_3_, Zn(NH_3_)_2_CS and others [45,47]. The studies conducted so far have shown that high efficiency of the process of removing heavy metals from wastewater from PCBs production is achieved with the use of Na_2_CS_3_ in the pH range of 9–9.5 [37,38,39]. Table 6 presents the calculated coefficients of the approximating polynomial for the experimental data presented in Table 1.

Consequently, the changes in the ∑_Cu,Cd,Zn_ value can be calculated according to the following formula:

∑_Cu,Cd,Zn_ (mg/L) = 52.71329 − 10.21092 (pH) + 0.52547 (pH)^2^ − 0.00910 (Na_2_CS_3_) + 0.00001 (Na_2_CS_3_)^2^ − 0.03449 (Time) + 0.00076 (Time)^2^ (9)

For optimal values of the three independent parameters (pH 9.72, Na_2_CS_3_ concentration 533.32 mg/L, Time 22.56 min), the sum of heavy metals in the treated wastewater (∑_Cu,Cd,Zn_ = 0.29 mg/L) was calculated and a verification experiment was performed. Under these conditions, the concentrations of Cu, Cd and Zn were 0.12 ± 0.01, 0.10 ± 0.01, and 0.05 ± 0.01 mg/L, respectively, and ∑_Cu,Cd,Zn_ = 0.27 ± 0.03 mg/L, as shown in Table 7. Interpretation of the obtained experimental and calculated values from the model for ∑_Cu,Cd,Zn_ (0.27 ± 0.03 versus 0.29 mg/L, respectively) requires the measurement uncertainty for the applied method of heavy metals determination in wastewater to be taken into account, which was ±10%. Therefore, the experimental value of ∑_Cu,Cd,Zn_ can vary in the range of 0.24–0.30 mg/L and means that the estimated value from the model (0.29 mg/L) is within the given concentration range of ∑_Cu,Cd,Zn_, which indicates the adequacy of the model.

Additionally, studies of Cu, Cd and Zn precipitation were carried out with the employment of conventional reagents used in sewage treatment plants. Due to the presence of Cd and the need for its quantitative precipitation, the wastewater was alkalinized to pH = 11 because the lowest concentration of Cd in treated wastewater is obtained only at pH approximately 11.2, and in the case of Cu and Zn at approximately 9.1 and 9.2 [55,66]. Under these conditions, using NaOH, Ca(OH)_2_ and CaO, a lower metal removal efficiency was achieved compared to the method with Na_2_CS_3_ (∑_Cu,Cd,Zn_ = 90.84, 93.97, 93.71 mg/L versus ∑_Cu,Cd,Zn_ = 99.69 mg/L, respectively). Effective precipitation of metals from wastewater required the use of Na_2_CS_3_ dose higher (by 7.2%) than the stoichiometric dose (533 versus 497 mg/L), which was probably related to the presence of chelating agents in the wastewater (as EDTA 180 mg/L) and the formation of complexes of heavy metals. Chelating agents reduce the efficiency of metal precipitation processes in the form of hydroxides [55,66] by the formation of stable complex compounds such as CuNa-NTA, ZnNa_2_EDTA, and others (where H_3_NTA is nitrilotriacetic acid, H_4_EDTA is ethylenediaminetetraacetic acid) [68]. The previous studies indicate that chelating agents enhance the difficulty of removing heavy metals, such as Cu, Zn, Co, Ni, Cd and Pb [69] and result in incomplete precipitation. Moreover, the increase in Zn concentration in treated wastewater, especially after NaOH application, may also be associated with the amphotericity of Zn(OH)_2_ and the formation of complex ions [Zn(OH)_4_]^2−^ [70,71]. Other investigators obtained a final concentration of Zn < 1 mg/L using Na_2_S together with chemical flocculant. They concluded that zinc removal by sodium sulfide is not affected by pH, that is an economic advantage as pH adjustment is not necessary [72]. Removal of chelated copper from wastewater by replacement precipitation by using ferrous salt was closely related to the molar ratio of Fe^2+^/Cu^2+^. When the Fe^2+^/Cu^2+^ ratio increased to 12, the Cu concentration in wastewater decreased from 25 to 0.38 mg/L, while the Cu^2+^/EDTA ratio in wastewater was 1:1 [73]. For Cd, application of coprecipitation with 100 mg/L FeCl_3_ at pH 9.0 removed 97% of Cd. The application of Al_2_(SO)_4_ instead of FeCl_3_ at pH 9.0 removed only 91.5% of Cd [74].

### 3.4. Toxicity Assessment

**Figure 4:**
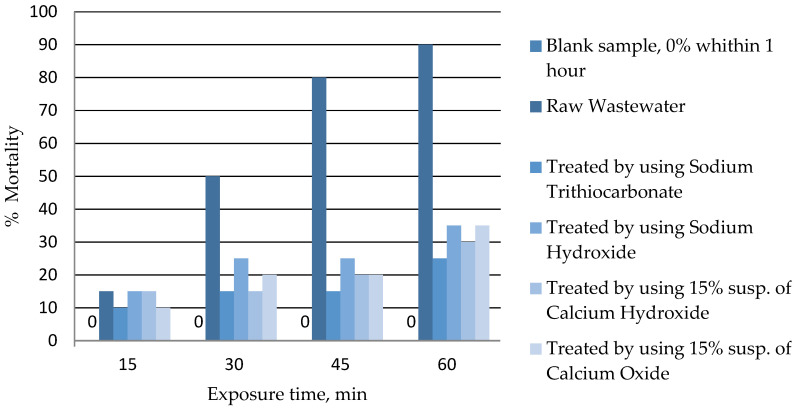
The results of the wastewater toxicity assessment against rotifer *B. plicatilis*.

Figure 4 shows the changes in the toxicity of wastewater to rotifer *B. plicatilis*, which is used to assess the toxicity of the marine environment [52]. Taking into account the significant salinity of galvanic wastewater, it is also useful for assessing the toxicity of the tested wastewater. The conducted tests showed that the use of Na_2_CS_3_ allowed the reduction in the toxicity of wastewater (expressed as percentage mortality of *B. plicatilis*) from 90% (untreated wastewater) to 25% (treated wastewater) after 60 min exposure time. In the case of conventional methods, the toxicity of treated wastewater was in the range of 30%–35%. Another study indicated the LC_50_ values (24 h) for *B. plicatilis* exposed to CuSO_4_ and CdSO_4_ to be 0.40 and 35.0 mg/L, respectively. These data show that *B. plicatilis* is more sensitive to Cu than to Cd by two orders of magnitude [75]. In the case of tested wastewater, reduction in toxicity is related to the removal of heavy metals, while final toxicity results from the presence of other compounds in the wastewater (including organic ones), that negatively affect the vital functions of *B. plicatilis*.

## 4. Conclusions

1. The use of Na_2_CS_3_ under optimal conditions determined with the use of CCD/RSM enables effective precipitation of heavy metals such as Cu, Cd and Zn from actual galvanic wastewater, even in the presence of complexing compounds.
2. The conventional methods of metal precipitation used so far, which consist of the alkalization of wastewater to the appropriate pH value in order to precipitate metal hydroxides, are less effective compared to the proposed methodology.
3. The use of rotifer *B. plicatilis* to assess the toxicity of treated wastewater indicated that it has decreased significantly, which is beneficial from an environmental point of view.
4. The application of Na_2_CS_3_ is not complicated and can be easily used in metal surface treatment plants.

Thus, Na_2_CS_3_ can be recommended as an effective compound to precipitate Cu, Cd and Zn from galvanic wastewater.

## Figures and Tables

**Table 1:** Empirical conditions for the Central Composite Design/Response Surface Methodology (CCD/RSM) and results (∑_Cu,Cd,Zn_ (mg/L)) for real galvanic wastewater.

Run	Experimental Conditions			Experimental Results *
	pH	Na_2_CS_3_ (mg)	Time (min)	∑_Cu,Cd,Zn_ (mg/L)
1	8.0	300	10	2.99 ± 0.30
2	8.0	300	30	2.79 ± 0.28
3	8.0	500	10	1.85 ± 0.19
4	8.0	500	30	1.70 ± 0.17
5	10.0	300	10	0.94 ± 0.09
6	10.0	300	30	0.90 ± 0.09
7	10.0	500	10	0.68 ± 0.07
8	10.0	500	30	0.57 ± 0.06
9	7.3	400	20	3.20 ± 0.32
10	10.7	400	20	0.80 ± 0.08
11	9.0	232	20	0.84 ± 0.08
12	9.0	568	20	0.67 ± 0.07
13	9.0	400	3	0.74 ± 0.07
14	9.0	400	37	0.72 ± 0.07
15 (C) **	9.0	400	20	0.75 ± 0.08
16 (C) **	9.0	400	20	0.76 ± 0.08

* Parameter value ± the measurement uncertainty for an extension factor *k* = 2; ** center of the plan.

**Table 2:** Determined physicochemical parameters of Na_2_CS_3_ solution.

Parameter	Unit	Result *
Color	−	Intense red
pH	−	>13
Specific density at 19 ℃	g/mL	1.319 ± 0.001
Na_2_CS_3_ content	%	40.8 ± 0.4

* Parameter value ± standard deviation.

**Table 3:** Determined physicochemical parameters of real galvanic wastewater.

Parameter	Unit	Result *
pH	−	3.4 ± 0.1
Electrical conductivity, EC	µS/cm	25,800 ± 1290
Salinity as NaCl	mg/L	12,850 ± 640
Complexing compounds as EDTA	mg/L	180 ± 28
Copper (Cu)	mg/L	59.0 ± 5.90
Cadmium (Cd)	mg/L	4.50 ± 0.45
Zink (Zn)	mg/L	22.70 ± 2.27

* Parameter value ± the measurement uncertainty for an extension factor *k* = 2; for pH, uncertainty was ±0.1%; for electrocoagulation (EC) and salinity, measurement uncertainty was ±5%; for complexing compounds, Cu, Cd, Zn was ±10%.

**Table 4:** Statistical parameters of the experiments using CCD/RSM—evaluation of the effects.

Parameter	Evaluation of the Effects, ∑_Cu,Cd,Zn_, mg/L, *R*^2^ = 0.9119, *R*^2^*_adj_* = 0.8532, 3 Parameters, 1 Block, 16 Experiments, MS = 0.1219								
	Effect	Standard Error	*p*-Value *	−95% Confidence Interval	+95% Confidence Interval	Factor	Standard Error of Factor	Lower Confidence Interval	Upper Confidence Interval
Constant Value	0.7197	0.2461	0.01693	0.1628	1.2765	0.7197	0.2461	0.1628	1.2765
pH (L)	−1.5049	0.1889	0.00002	−1.9324	−1.0775	−0.7525	0.0945	−0.9662	−0.5388
pH (Q)	1.0509	0.2294	0.00133	0.5320	1.5699	0.5255	0.1147	0.2660	0.7850
Na_2_CS_3_ (L)	−0.4548	0.1889	0.03942	−0.8823	−0.0274	−0.2274	0.0945	−0.4411	−0.0137
Na_2_CS_3_ (Q)	0.1706	0.2294	0.47608	−0.3484	0.6896	0.0853	0.1147	−0.1742	0.3448
Time (L)	−0.0781	0.1889	0.68885	−0.5056	0.3493	−0.0391	0.0945	−0.2528	0.1746
Time (Q)	0.1529	0.2294	0.52178	−0.3660	0.6719	0.0765	0.1147	−0.1830	0.3359

L—linear effect; Q—quadratic effect; * statistically significant if *p* < 0.05. The analysis showed a significant (*p* < 0.05) influence of pH (L), pH (Q) and Na_2_CS_2_ (L) on the value of ∑_Cu,Cd,Zn_. Other parameters turned out to be statistically insignificant (*p* > 0.05), including Time (L) and Time (Q). The calculated values of *R*^2^ = 0.9119 and *R*^2^_adj_ = 0.8532 (91.19% and 85.32%, respectively) indicated that 91.19% of the variability of the dependent variable was explained by the model.

**Table 5:** Analysis of the CCD/RSM—verification of the adequacy of the model using analysis of variance (ANOVA).

Parameter	Assessment of Effects, ∑_Cu,Cd,Zn_, mg/L, *R*^2^ = 0.9119, *R*^2^*_adj_* = 0.8532, 3 Parameters, 1 Block, 16 Experiments, MS = 0.1219			
	SS	MS	F	*p*-Value *
pH (L)	7.7326	7.7326	63.4384	0.00002
pH (Q)	2.5580	2.5580	20.9860	0.00133
Na_2_CS_3_ (L)	0.7064	0.7064	5.7950	0.03942
Na_2_CS_3_ (Q)	0.0674	0.0674	0.5530	0.47608
Time (L)	0.0209	0.0209	0.1711	0.68885
Time (Q)	0.0542	0.0542	0.4443	0.52178
Error	1.0970	0.1219	-	-

L—linear effect, Q—quadratic effect, SS—predicted residual error of sum of squares, MS—mean square error, F—statistics and * statistically significant if *p* < 0.05.

**Table 6:** Coefficients of the fitted model *.

Predictor	Regression Coefficient	Standard Error	t−Value, df ** = 9	*p*–Value ***	−95% Confidence Interval	+95% Confidence Interval
Intercept	52.71329	10.26366	5.13592	0.00061	29.49528	75.93130
pH (L)	−10.21092	2.06685	−4.94032	0.00080	−14.88646	−5.53537
pH (Q)	0.52547	0.11471	4.58105	0.00133	0.26599	0.78495
Na_2_CS_3_ (L)	−0.00910	0.00922	−0.98623	0.34979	−0.02997	0.01177
Na_2_CS_3_ (Q)	0.00001	0.00001	0.74361	0.47608	−0.00002	0.00003
Time (L)	−0.03449	0.04684	−0.73627	0.48032	−0.14046	0.07148
Time (Q)	0.00076	0.00115	0.66655	0.52178	−0.00183	0.00336

* represent the fifth decimal place; ** df—degree of freedom; *** statistically significant if *p* < 0.05.

**Table 7:** Selected physicochemical parameters of galvanic wastewater before and after treatment.

Parameter *	Before Treatment	After Treatment by Using Na_2_CS_3_ (pH 9.7, Na_2_CS_3_ 533 mg/L, Time 23 min) **	Removal, % ***	After Treatment by Using NaOH (Alkalization up to pH 11)	Removal, % ***	After Treatment by Using 15% Suspension of Ca(OH)_2_ (Alkalization up to pH 11)	Removal, % ***	After Treatment by Using 15% Suspension of CaO (Alkalization up to pH 11)	Removal, % ***
pH	3.4 ± 0.1	9.7 ± 0.1	-	11.0 ± 0.1	-	11.1 ± 0.1	-	11.1 ± 0.1	-
Copper (Cu), mg/L	59.0 ± 5.90	0.12 ± 0.01	99.80	2.50 ± 0.25	95.76	2.9 ± 0.29	95.08	2.8 ± 0.28	95.25
Cadmium (Cd), mg/L	4.50 ± 0.45	0.10 ± 0.01	97.78	0.8 ± 0.08	82.22	0.50 ± 0.05	88.89	0.42 ± 0.04	90.67
Zink (Zn), mg/L	22.70 ± 2.27	0.05 ± 0.01	99.78	4.6 ± 0.46	79.74	2.1 ± 0.21	90.75	2.2 ± 0.22	90.31
∑_Cu,Cd,Zn,_ mg/L	86.20 ± 8.62	0.27 ± 0.03	99.69	7.90 ± 0.79	90.84	5.50 ± 0.55	93.97	5.42 ± 0.54	93.71

* Parameter value ± the measurement uncertainty for an extension factor *k* = 2; for pH, the uncertainty was ±0.1; for Cu, Cd, Zn the uncertainty was ±10%; ** in optimal conditions; *** Removal = $\frac{\left(C1-C2\right)\;\times \;100\%}{C1}$, where *c*_1_—concentration of metal in raw wastewater, *c*_2_—concentration of metal in treated wastewater.

## Data Availability

Data sharing not applicable.
